# Supplementary material for: Sulfolobus acidocaldarius Microvesicles Exhibit Unusually Tight Packing Properties as Revealed by Optical Spectroscopy
Source: Int J Mol Sci. 2019 Oct 25;20(21):5308. doi: 10.3390/ijms20215308 (PMC6862217; doi:10.3390/ijms20215308)
Supplement: Supplementary file 1 [file ijms-20-05308-s001.pdf]

Supplemental materials

Sulfolobus acidocaldarius Microvesicles Exhibit Unusually Tight Packing Properties as Revealed by Optical Spectroscopy

Alexander Bonanno[a], Robert C. Blake II[b], and Parkson Lee-Gau Chong[a,*]
[a] Department of Medical Genetics and Molecular Biochemistry, The Lewis Katz School of Medicine at Temple University, Philadelphia, Pennsylvania 19140, USA, and [b] College of Pharmacy, Xavier University of Louisiana, New Orleans, Louisiana, 70125, USA

*Corresponding authors: Parkson Chong, email address: pchong02@temple.edu

Table S1. Steps involved in the isolation of Sa-MVs from the cell suspensions [8]

Step	Method; Rotor/Centrifuge	G force	rpm	Time (min)	Collect
1	Sorvall SLA-3000/ Sorvall RC5B Plus	11,000 x g	8,000	20	supernatant
2	ultrafiltration				concentrated supernatant
3	Sorvall T-875/ Beckman XL-90	126,000 x g	36,000	45	¼ supernatant and pellet
4	Sorvall T-875/ Beckman XL-90	19,000 x g	14,000	20	supernatant
5	Sorvall T-875/ Beckman XL-90	374,000 x *g*	62,000	60	Pellet (i.e., Sa-MVs)

Table S2. Dialysis does not change particle size and PDI of Sa-MAs

Time Dialyzed (hrs)	Particle Size (nm)	PDI
**0**	213.40 ± 0.79	0.187 ± 0.030
**4**	209.70 ± 1.57	0.176 ± 0.006
**8**	209.43 ± 1.97	0.185 ± 0.006
**12**	210.57 ± 1.63	0.185 ± 0.005
**16**	208.40 ± 2.46	0.186 ± 0.007
**20**	210.27 ± 2.54	0.192 ± 0.016
**24**	211.77 ± 6.09	0.179 ± 0.027
**28**	209.07 ± 2.76	0.181 ± 0.012

Table S3. Effect of temperature on the emission maximum of Sa-MV intrinsic fluorescence in 50 mM pyrophosphate buffer. Excitation wavelength = 275 nm. Red: heating mode; blue: cooling mode. Like the case of Sa-MVs in Tris buffer, a blue shift in $\lambda_{em,max}$ of 8 nm is seen in the intrinsic protein fluorescence when the temperature is changed from 20 to 52.2°C.

Temperature (˚C)	Emission Maximum (nm)
20.0	303.0
24.0	300.0
28.2	298.0
34.7	298.0
47.3	298.0
52.2	295.0
48.0	296.0
34.8	298.0
20.5	303.0

Table S4. The particle sizes and PDIs of Sa-MVs and various liposomes used in the Laurdan fluorescence measurements are tabulated below. Sizes and PDIs were determined at 25°C.

	Size (nm)	PDI
LUV_{POPC}	199.9 ± 3.0	0.102 ± 0.017
LUV_{DPPC}	202.9 ± 6.6	0.275 ± 0.030
LUV_{PLFE}	195.6 ± 3.3	0.115 ± 0.015
Sa-MV	199.9 ± 0.2	0.195 ± 0.036
LUV_{MV}	218.6 ± 2.3	0.244 ± 0.026

Table S5. The REES values (in the unit of nm) obtained from Sa-MVs and various liposomes at four different temperatures

Temperature (˚C)	LUV_{POPC}	LUV_{DPPC}	LUV_{PLFE}	Sa-MV	LUV_{MV}
18.0	2.81 ± 0.23	2.21 ± 0.28	12.56 ± 0.47	18.94 ± 0.27	14.03 ± 0.43
36.0	2.01 ± 0.22	1.75 ± 0.14	11.12 ± 0.25	16.18 ± 0.07	12.71 ± 0.44
56.0	--	4.62 ± 0.16	9.48 ± 0.16	15.14 ± 0.14	10.38 ± 0.38
66.7	0.36 ± 0.17	5.04 ± 0.07	9.33 ± 0.33	14.51 ± 0.24	10.16 ± 0.46
